# Impact of thermal frequency drift on highest precision force microscopy using quartz-based force sensors at low temperatures

**DOI:** 10.3762/bjnano.5.48

**Published:** 2014-04-04

**Authors:** Florian Pielmeier, Daniel Meuer, Daniel Schmid, Christoph Strunk, Franz J Giessibl

**Affiliations:** 1 Institute of Experimental and Applied Physics, University of Regensburg, D-93053 Regensburg, Germany

**Keywords:** AFM, frequency drift, length extensional resonator, needle sensor, qPlus sensor, quartz

## Abstract

In frequency modulation atomic force microscopy (FM-AFM) the stability of the eigenfrequency of the force sensor is of key importance for highest precision force measurements. Here, we study the influence of temperature changes on the resonance frequency of force sensors made of quartz, in a temperature range from 4.8–48 K. The sensors are based on the qPlus and length extensional principle. The frequency variation with temperature *T* for all sensors is negative up to 30 K and on the order of 1 ppm/K, up to 13 K, where a distinct kink appears, it is linear. Furthermore, we characterize a new type of miniaturized qPlus sensor and confirm the theoretically predicted reduction in detector noise.

## Findings

Frequency modulation atomic force microscopy [1] has become an essential tool for surface scientist‘s to study chemical and magnetic interactions at the atomic scale [2–6]. In FM-AFM the frequency shift Δ*f* = *f* – *f*_0_ of a mechanical oscillator with stiffness *k* upon tip–sample interaction is measured, while the oscillation amplitude *A* is kept constant. For quantitative force measurements the uncertainty in the force gradient is crucial [7]. Frequency shift and force gradient are related via

[1]
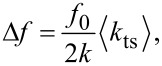


where 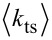 is the averaged force gradient between tip and sample, which can be deconvolved into *k*_ts_ [8]. Four noise contributions limit the accuracy of the Δ*f* measurement, which are inherent to FM-AFM [7]. Deflection detector noise [1,9–11] is proportional to the measurement bandwidth *B* with *B*^1.5^, thermal [1] and oscillator noise [11] are proportional to *B*^0.5^. At higher bandwidths (e.g., *B* > 100 Hz), deflection detector noise is usually the dominant noise contribution [10]. If the measurement bandwidth *B* can be set sufficiently small, e.g., at low temperatures, these noise contributions are significantly reduced and imaging with millihertz resolution becomes possible [12]. In turn, when *B* is small the stability of the eigenfrequency *f*_0_ is particularly important, because frequency drift noise is proportional to 1/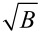 [7]. The main cause of frequency drift are changes in *f*_0_ with temperature *T*, which are material dependent. Even for experiments conducted at liquid helium temperatures, temperature drift limits the achievable resolution. Changes in ambient pressure affect the boiling temperature of helium, e.g., the vapor pressure of He^4^ at 4.4 K changes at a rate of ≈10^5^ Pa/K [13]. Typical changes in ambient pressure are between 100–500 Pa/day, leading to temperature changes in the range of 1–5 mK/day.

**Figure 1:**
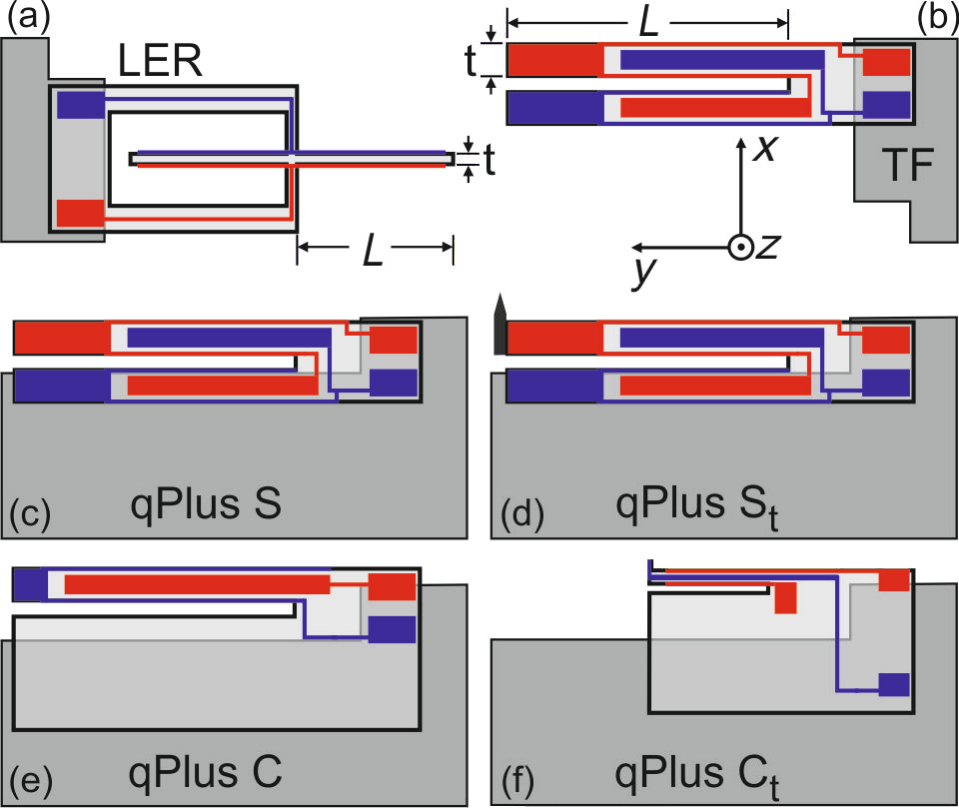
(Color online). Geometry of length extensional resonator (a), tuning fork (b), standard qPlus sensors (c,d) and custom made qPlus sensors (e,f). The two coupled oscillators (LER and TF) are fixed to the substrate at their base, both prongs oscillate, no additional mass or tip is attached. Standard qPlus sensors (S and S_t_) are based on quartz TFs, one prong is fixed to the substrate, in (d) a tip is added to the free prong. Custom designed qPlus sensors consist only of a single prong with a larger base, which is fixed to the substrate, unnecessary electrodes are removed to reduce capacity. The beam dimensions of sensor C (e) are the same as for sensors S and S_t_. Sensor C_t_ (f) has a shorter and thinner beam, see Table 1 for details. Note, the *z*-axis (optical axis) is not exactly perpendicular to the oscillation of the beams, but off by ≈2°.

Since the introduction of the AFM by Binnig et al. [14] mainly force sensors made of silicon are in use [10]. In the last decade force sensors based on quartz resonators became more attractive, with quartz tuning forks (TF) in the “qPlus” configuration (Figure 1c–f) [15] and length extensional resonators (LER) as the so called “needle sensor” (Figure 1a) [16]. Quartz resonators are usually designed and characterized for room temperature applications. Their remarkable frequency stability in comparison to silicon cantilevers results in a significantly smaller frequency drift at room temperature [7,10]. The frequency variation with temperature resembles an inverted parabola centered around the turnover temperature, which is usually tuned to about 25 °C [17]. On the other hand, quartz-based force sensors are often used in a low temperature environment, but little is known about the frequency variation with temperature in this regime.

Hembacher et al. evaluated the relative frequency change ε = δ*f*/*f*_0_ of an encapsulated quartz TF over a large temperature range from 300 K down to 4.2 K, where ε decreases monotonically with *T* [18]. At 300 K and 4.2 K ε is almost zero, hence the influence of temperature variations should be minimized here. In a more detailed measurement, Rychen et al. measured the frequency change of a quartz TF from 1.5 K to 50 K at a constant pressure of 10 mbar, here *f*_0_ is not increasing monotonically with *T* but shows a local minimum around 20 K [19]. This resembles qualitatively the temperature dependence of the Grüneisen parameter γ, which relates thermal expansion to vibrational properties [20]. The calculated values for γ show a maximum around 30 K decreasing sharply to lower temperatures and gradually to higher ones [20–21]. Additionally the anisotropic thermal expansion coefficients of quartz, α_||_ and 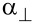, parallel and perpendicular to the optical axis also show a non linear behavior with temperature [22]. Here, 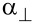 increases monotonically with *T*, whereas α_||_ is negative below ≈12 K [21].

The eigenfrequency *f*_0_ of a beam oscillating in a bending mode is given via

[2]
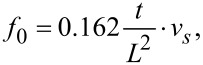


where *L* is the length, *t* the thickness of the beam, and 
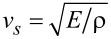
 the velocity of sound with *E* being Young‘s modulus and ρ mass density of quartz. In case of the LER one obtains [7]

[3]
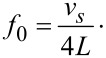


Hence, *f*_0_ changes, when the dimensions of the beam or *v_s_* changes due to thermal expansion. The orientation of the beams of the quartz sensors deviate slightly from a perpendicular orientation to the optical axis (*z*-axis, Figure 1). This is due to the crystal cut, which is not exactly along the optical axis, but off by about 2° (+2° *X*-cut). Hence, the direction of *L* is off by 2° and *t* is perpendicular to the optical axis. The thermal expansion along these directions is mainly determined by 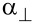, which increases monotonically from 4 K up to room temperature [21]. Neglecting this small deviation in *L* direction one obtains from Equation 2 for the frequency change with temperature of TF and qPlus sensors

[4]
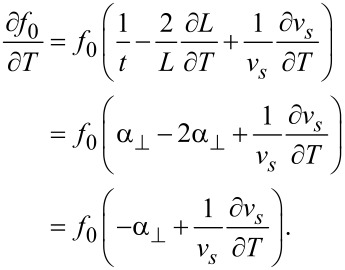


The same result is obtained for the LER geometry. For *X* cut crystalline quartz no change in *v_s_* within a precision of 0.1 ppm was observed below 10 K [23–25]. The measured values of 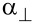 below 10 K are in the order of 0.01 ppm/K [21]. According to Equation 4, the variation of ε with *T* is therefore expected to be in the range of 0.1 ppm/K below 10 K and similar for TF, qPlus and LER geometry.

In this work, we measure the frequency change with temperature from 4.8 K to 48 K for quartz based force sensors. Six different quartz resonators were investigated to directly evaluate and compare the influence of thermal frequency drift on the force gradient noise. Two coupled oscillators, a LER (Figure 1a) and a TF (Figure 1b) both without tip, were used for direct comparison. Two standard qPlus sensors were built with quartz TFs, one without tip (S, Figure 1c) and one with a tip (S_t_, Figure 1d). Finally, custom designed quartz cantilevers, are used to build qPlus sensors with standard and smaller beam dimensions (C and C_t_, Figure 1e, Figure 1f). At the end of the prong of sensor C_t_ is a small appendix for easier accommodation of tips, which acts effectively as an additional mass, see Figure 1f. The advantages of the custom designed sensors in contrast to the standard qPlus sensors will be briefly discussed at the end of this letter. In Table 1 the relevant parameters of the sensors are summarized. All quartz resonators were glued with non-conductive epoxy to an aluminum oxide substrate which is commonly used for our qPlus sensors, the electrodes are then contacted with conductive epoxy. For the low temperature measurements the substrates where glued again with non-conductive epoxy onto a piece of copper. The copper piece serves as a thermal anchor and can be mounted on a He^4^ stick, usually used for transport measurements. The stick is equipped with a heater resistor and a Si diode to measure *T*. The sensors were excited electrically with a constant amplitude *A* and the deflection signal was measured with a commercial charge amplifier [26]. Finally, the frequency shift was determined by a digital phase locked loop stabilized by an oven-controlled quartz resonator with a precision of 1 ppb/day [27]. For the measurements, the temperature setpoint was increased at a rate of 0.5 K/min and the change in eigenfrequency was monitored. At this rate the maximum deviation of the actual temperature from the setpoint temperature was below 0.1 K.

**Table 1:** Dimensions (length *L*, thickness *t* and width *w*), resonance frequency *f*_0_ and stiffness *k* of the different types of sensors investigated. The highest *Q* values are obtained with the custom qPlus sensors. The values for *f*_0_ and *Q* are obtained from resonance curves measured at 4.2 K.

	*L* (μm)	*t* (μm)	*w* (μm)	*f*_0_ (Hz)	*k* (N/m)	*Q*

qPlus S	2400	214	130	32680	1800	264000
qPlus S_t_	2400	214	130	19658	1800	179000
qPlus C	2400	214	130	32884	1800	397000
qPlus C_t_	992	85	145	73303	1830	312000
TF	2400	214	130	32742	1800	140000
LER	1340	70	130	998148	530000	202000

**Figure 2:**
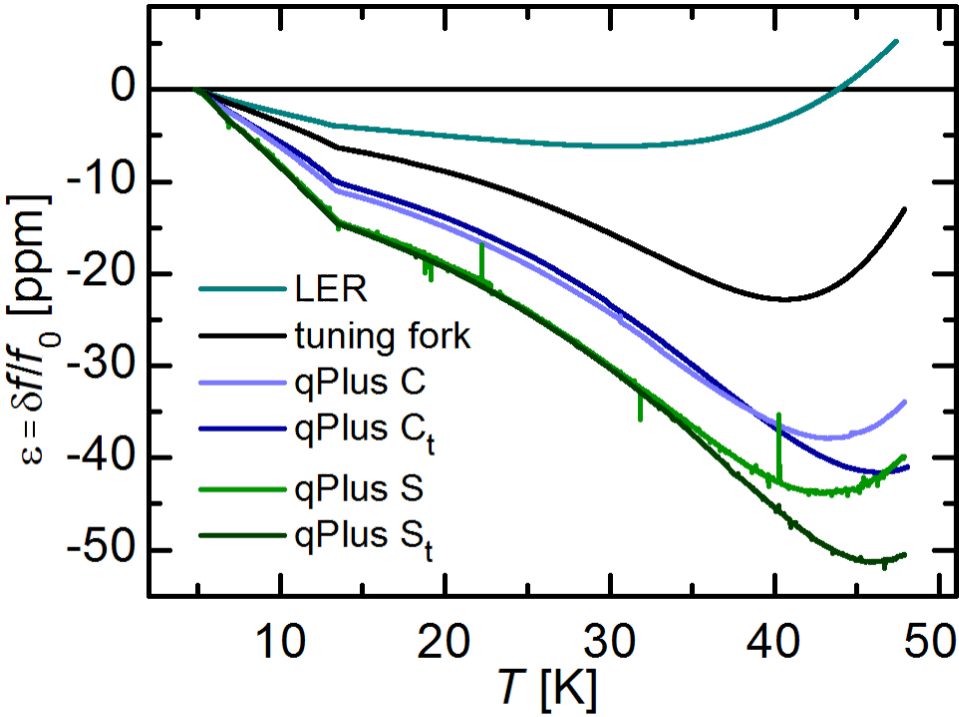
(Color online). Relative frequency change of quartz based AFM sensors from 4.8–48 K. The coupled oscillators show less relative frequency change with temperature as the qPlus sensors. For custom made qPlus sensors ε is smaller as for the standard ones. The spikes in the curves of the qPlus sensors arise from mechanical excitations of the sensors due to external vibrations or sound.

The results of the low temperature measurements are shown in Figure 2, where the relative frequency change ε is plotted against *T*. As the He^4^ stick is not equipped with a vibration isolation system, there are some sharp peaks in the curves for the qPlus sensors caused by mechanical excitations of the sensors. This is not an issue for the coupled oscillators, and also for the sensor C_t_, which has a higher resonance frequency and is therefore less affected by external vibrations. Obviously, there is a difference in ε for the various types of sensors used. Overall, the relative change is smallest for the LER followed by the TF and the custom qPlus sensors (C, C_t_), the standard qPlus sensors (S, S_t_) show the strongest change of *f*_0_ with *T*. The curves for S and S_t_ lie exactly on top of each other and start to split up at around 33 K. For the two custom sensors ε is also quite similar and they split up at around 40 K. For sensors S and C, without an additional mass at the end of the prong, ε changes its sign earlier than in case of S_t_ and C_t_. All curves show a fairly linear decrease of ε up to 13 K where a distinct kink appears, which might be due to the sign change in α_||_. After this kink, ε still decreases for all types of sensors. In case of the LER the sign of *∂*ε/*∂T* changes from negative to positive at around 30 K, this agrees qualitatively with the temperature dependence of the Grüneisen parameter γ. For tuning fork based sensors, ε still decreases, and *∂*ε/*∂T* changes its sign at temperatures between 40–47 K.

In the temperature range from 5–12 K the slopes η can be obtained from a linear fit to the data in Figure 2. The determined values for η are all in the order of 1 ppm/K and summarized in Table 2. This is much higher than expected from the change in *v_s_* or 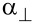, as discussed above. From neutron irradiation it is known, that the change of *v_s_* with *T* depends strongly on the quality of the crystal, the rate of change increases linearly with the defect density [24–25,28]. Because the quartz resonators studied in this work are not from the same wafer and manufacturer, one might argue that the differences in *∂*ε/*∂T* can be caused by different crystal quality and material processing. But there are two important observations from the measurements presented in Figure 2, which are contradictory to that. First, the difference between the standard qPlus sensors (S, S_t_) and the tuning fork is somewhat unexpected, because tuning forks from the same batch were used to build these sensors. Second, the striking similarity between sensors C and C_t_, which have different beam dimensions and are not even from the same manufacturer. Hence, the influence of different crystal quality or material processing on the frequency variation with temperature, is expected to be largest for these two types of sensors. Obviously, this is not the case and suggests that the difference between the tuning fork and the standard qPlus sensors is due to the different geometry and the mechanical coupling of the beam to the support. This could also explain the smaller values of ε and η for the coupled oscillators, because they are less influenced by the mechanical coupling of the quartz oscillator to the support than the qPlus sensors.

For qPlus sensors, the resonance frequency *f*_0_ can also be expressed as 
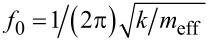
, where *m*_eff_ = 0.24 m is the effective mass of the oscillating prong. Hence, *∂*ε/*∂T* can be interpreted as a variation of the stiffness with temperature, implying *m*_eff_ remains unchanged. The similarity of *∂*ε/*∂T* for sensors S and S_t_ up to a temperature of around 33 K, leads to the conclusion that there is no significant influence of the additional mass of the tip on the effective stiffness of sensor S_t_, compared to sensor S.

**Figure 3:**
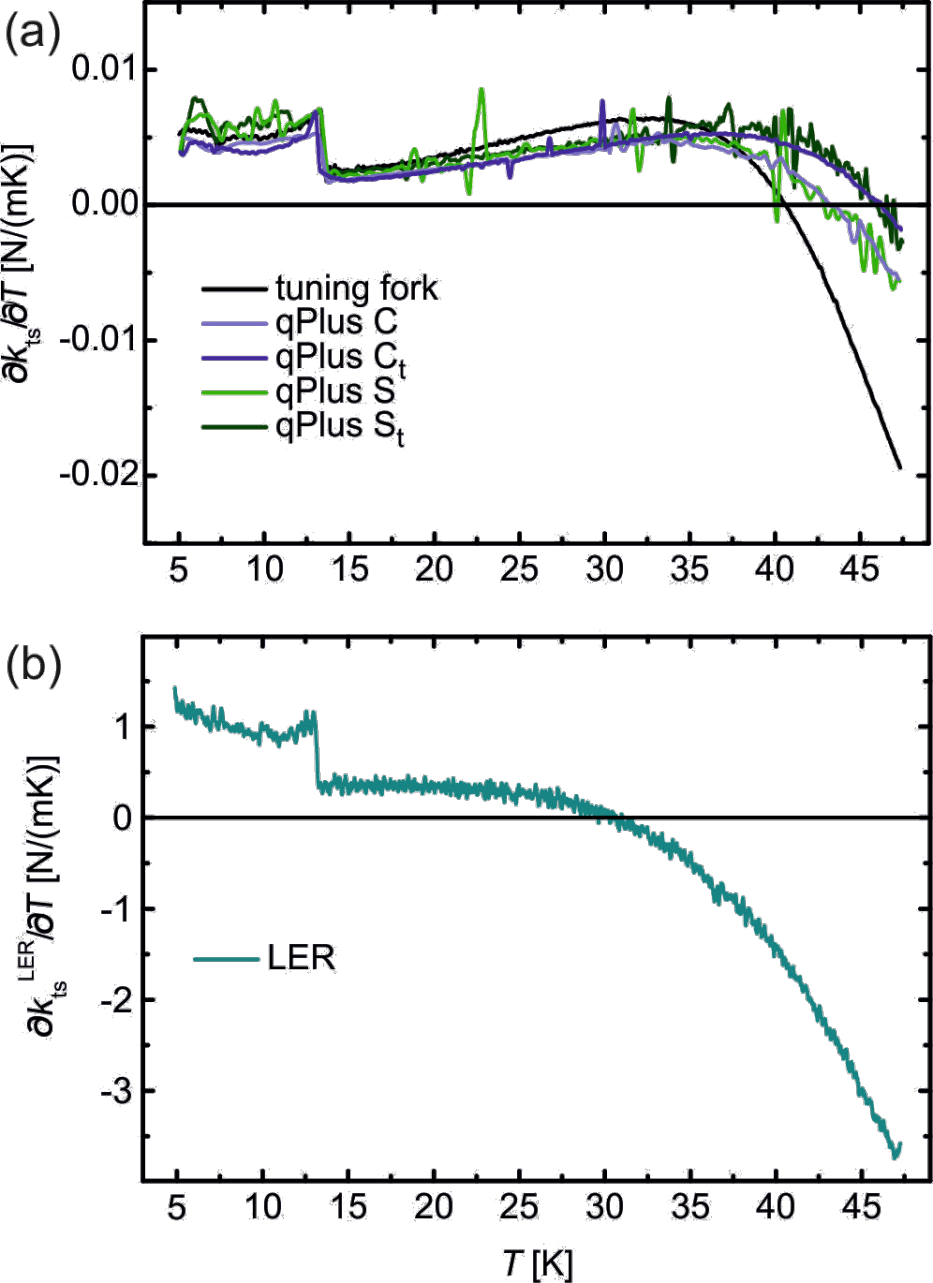
(a) Numerical derivative *∂k*_ts_/*∂T* for all tuning fork and qPlus sensors from 5–47 K. (b) *∂k*_ts_/*∂T* for LER. Note, the *y* scale is different for (a) and (b).

So far, only the relative frequency change with temperature was discussed. The influence on the measured force gradient *∂k*_ts_/*∂T* is obtained by multiplying the slopes η with the according value of 2 × *k* [7]. The values for *∂k*_ts_/*∂T* are also given in Table 2, due to the much higher stiffness of the LER, *∂k*_ts_/*∂T* is more than two orders of magnitude higher than in case of the tuning fork and qPlus sensors. In a previous study we have already discussed the influence of thermal frequency drift theoretically [7], there the frequency drift for LER and qPlus sensors was assumed to be about 1 ppm/K as estimated from Figure 2a in [19]. Actually, the LER shows only about half of this value whereas for qPlus sensors the frequency drift is about a factor of 1.5 higher. Resulting in a force gradient drift noise, which scales with *k*, that is 160–240 times higher for LER sensors than for tuning fork or qPlus sensors. This is illustrated in Figure 3, where *∂k*_ts_/*∂T* is displayed for the tuning fork and the qPlus sensors (Figure 3a) and the LER (Figure 3b). Again, the wiggles in the curves for the qPlus sensors are caused by external excitations due to a lacking damping system. The kink around 13 K from Figure 2 shows up as a clear step. In temperature dependent measurements it might therefore be beneficial for a stable operation of the force sensor to avoid temperatures around 13 ± 0.5 K.

**Table 2:** Slopes η from linear fits to the data in a temperature range from 5–12 K. For conversion of frequency shift to force gradient, the corresponding *k* values from Table 1 were used, in case of TF and LER the stiffness was multiplied with a factor of 2 [7].

	η(ppm/K)	*∂k*_ts_/*∂T* (mN/m/K)

qPlus S	−1.69	6.1
qPlus S_t_	−1.63	5.9
qPlus C	−1.25	4.5
qPlus C_t_	−1.11	4.3
TF	−0.70	5.0
LER	−0.46	960

In the last part, the benefits of the custom designed qPlus sensors C and C_t_ are briefly discussed. They exhibit higher and more reliable *Q* values, this is attributed to the larger base of the sensor. The clamping point of the beam, where the mechanical strain is highest, is now further away from the glued part as in case of tuning fork based qPlus sensors, see Figure 1. With the qPlus sensors of type C_t_, *Q* values exceeding 1,300,000 have been achieved [6]. The design of sensor C_t_ is based on an analysis of the signal-to-noise ratio of quartz sensors, which showed that deflection detector noise decreases with decreasing beam thickness *t* [7]. While reducing simply *t* would lead to a decrease in *k* as well, the length *L* was also decreased to keep *k* in the optimal stiffness range for atomic resolution imaging [29]. These custom made sensors have now length *L* = 0.922 mm and thickness *t* = 85 μm. On the upper and lower side of the prong are etched grooves which lead to a more idealized electric field distribution [7]. For quartz-based force sensors, detector noise is proportional to

**Figure 4:**
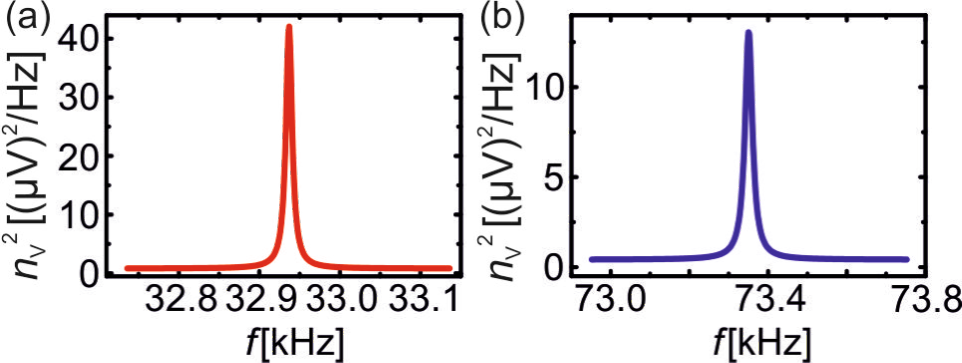
Thermal excitation spectra for sensors C (a) and C_t_ (b). The center frequency in (a) is *f*_0_ = 32.939 kHz and the frequency range is 400 Hz, in (b) *f*_0_ = 73.351 kHz and the range is 800 Hz. The sensitivities, determined from the area under the peaks, are *S*^C^ = 16.3 μV/pm and 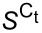 = 14.1 μV/pm. The electrical noise densities are 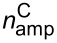 = 892 nV/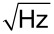 and 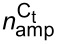 = 636 nV/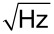, resulting in 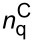 = 55 fm/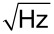 and 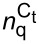 = 45 fm/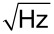.

[5]
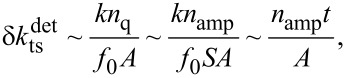


where *n*_q_ = *n*_amp_/*S* is the deflection noise density, *n*_amp_ the electrical noise density of the preamplifier, *S* the sensitivity of the sensor and *A* the oscillation amplitude [7]. For fixed values of *n*_amp_ and *A* one expects an improvement for sensor C_t_ in detector noise by a factor of 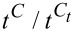 = 214 μm/85 μm = 2.5. Before we can compare 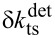 for sensors with different beam thickness we have to determine the stiffness *k* for the custom designed sensor C_t_. This is done by measuring its resonance frequency *f*_0_ at room temperature and calculate *k* from *k* = (2π*f*_0_)^2^(*m*_eff_ + *m*_app_), where *m*_app_ is the mass of the appendix at the end of the prong. The masses are calculated via *m* = ρ*V*, where ρ is the mass density of quartz and the volume *V* is determined by measuring the dimensions of the beam with an optical microscope. Finally, taking the additional mass *m*_app_ at the end into account gives a stiffness of *k* = 1830 N/m with a relative error of ±10%. For the determination of *n*_q_ one can use thermal excitation spectra of the sensors C and C_t_ at room temperature analogous to previous studies [7,15,30]. For this purpose the sensors C and C_t_ were put into a metal box for shielding, one of the electrodes was grounded, whereas the second electrode was connected via a BNC feedthrough to the charge amplifier [26]. The output signal of the charge amplifier was measured with a spectrum analyzer (Agilent 35670A Dynamical Analyzer). The measured thermal excitation spectra at room temperature are shown in Figure 4. From there, the values of *n*_q_ for sensors C and C_t_ are determined as 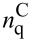 = 55 fm/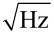 and 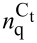 = 45 fm/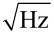. With the values for *k* from Table 1 and *f*_0_ from Figure 4 for sensors C and C_t_ the ratio of the deflection noise is given as 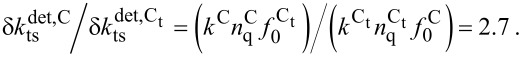 The reduction in detector noise by a factor of 2.7 is even more than the expected value of 2.5 from the decrease in *t*. We attribute this is to a better performance (lower *n*_amp_) of the preamplifier at higher resonance frequencies.

In summary, the variation of *f*_0_ with *T* for qPlus, tuning fork and LER sensors was measured at low temperatures and the resulting influence on the force gradient noise was determined. For temperature changes in the order of 1mK the minimum detectable force gradient is about 1 mN/m for the LER and about 5 μN/m for qPlus sensors. Furthermore, the decreased deflection detector noise of custom qPlus sensors of type C_t_ was discussed, which is due to the reduced thickness *t* of the beam.
